# Decision‐making between DCA and VMAT in Linac‐based SRS for brain lesions: A dosimetric analysis based on tumor size and geometry and introduction of Qasym

**DOI:** 10.1002/acm2.70282

**Published:** 2025-10-21

**Authors:** Lara Caglayan, Davide Scafa, Patrick Eich, Christina Leitzen, Fabian Kugel, Stephan Garbe, Gustavo Renato Sarria, Christos Moustakis, Dimos Baltas, Julian Philipp Layer, Cas Stefaan Dejonckheere, Andrea Renate Glasmacher, Shari Wiegreffe, Anca L Grosu, Eleni Gkika, Youness Nour

**Affiliations:** ^1^ Department of Radiation Oncology University Hospital Bonn Bonn Germany; ^2^ Department of Radiation Oncology University of Leipzig Medical Center Leipzig Germany; ^3^ Department of Radiation Oncology Universitätsklinikum Freiburg Freiburg im Breisgau Germany; ^4^ Institute of Experimental Oncology University Hospital Bonn Bonn Germany

**Keywords:** brain metastases, dosimetric comparison, dynamic conformal arc (DCA), lesion asymmetry, Qasym, stereotactic radiosurgery (SRS), treatment planning optimization, volumetric modulated arc therapy (VMAT)

## Abstract

**Introduction:**

Linac‐based SRS provides a highly precise noninvasive treatment option for intracranial lesions. DCA and VMAT are commonly used Linac‐based techniques. There are no standardized guidelines for technique selection, particularly considering the geometric properties of the lesions. We thus sought to compare DCA and VMAT to define a workflow for clinical decision‐making. This study introduces Q_asym_, a novel parameter quantifying lesion asymmetry, and compares the dosimetric performance of DCA and VMAT.

**Materials and methods:**

Between 2018 and 2021, a total of 89 brain metastases from 24 patients with lesion volumes up to 4.3 cm^3^ were included. VMAT and DCA plans were created for each lesion with identical field parameters, resulting in a total of 178 evaluable plans. The parameters included the CI, DSI, and MUs. Various approaches for quantifying asymmetry were explored and their impact on CI and DSI was investigated. Additionally, we introduced the novel Q_asym_ index, designed to quantify lesion asymmetry.

**Results:**

VMAT resulted in lower CI values, especially for volumes exceeding 2 cm^3^. With a prescription dose of 20 Gy, DSI_90%_ values were comparable between VMAT and DCA for < 2 cm^3^, while VMAT achieved lower values for larger volumes. VMAT showed higher DSI_50%_ and DSI_25%_ values for < 2 cm^3^, which reversed for larger volumes. V_2Gy_ values were similar for both techniques for < 2 cm^3^ but were lower for VMAT in larger volumes. Q_asym_ significantly correlated with CI (*p* < 0.01). Analysis of ΔCI% and ΔDSI% revealed that VMAT outperformed DCA for lesions > 2 cm^3^ and smaller volumes with a Q_asym_ > 1.2. DCA required significantly fewer MUs (*p* < 0.01).

**Conclusion:**

This study provides detailed dosimetric information on two commonly applied planning techniques for Linac‐based SRS treatment for brain metastases. These findings might support decision making for optimal technique selection upon volumetric features. Q_asym_ provides a practical tool for these purposes.

## 1 INTRODUCTION

Stereotactic radiosurgery (SRS) represents a highly precise form of radiation therapy that has proven effective, particularly in the treatment of intracranial lesions. Its applications span from malignant brain tumors^1^, ^2^, ^3^ to arteriovenous malformations,^4^ vestibular schwannomas,^5^ and intraocular melanomas. The use of SRS serves as an alternative or complement to invasive surgery, without compromising local tumor control^6^ while patients may benefit from noninvasive treatment and reduced in‐hospital times. Side effects from SRS are gaining importance regarding quality of life, as patients tend to survive longer due to newer systemic treatments and might potentially experience more adverse events during follow‐up.^7^, ^8^

Frequently, for Linac‐based SRS, either dynamic conformal arc (DCA) or volumetric modulated arc‐therapy (VMAT) techniques are employed. Both techniques have specific characteristics and dose adjustment strategies. DCA utilizes a radiation arc or multiple non‐coplanar radiation arcs. During this process, the multi‐leaf collimator (MLC) leaves are dynamically adjusted to conform to the (sometimes irregular) target contour, allowing for rather conformal and less homogeneous radiation delivery. In contrast, VMAT generates fluence modulation by continuously moving the MLC leaves, adjusting the gantry speed, and varying the dose rate, resulting in improved dose conformity.^2^ The use of VMAT is currently recommended for patients with multiple lesions.^2^ However, there are currently no uniform guidelines addressing the selection of the most suitable technique while considering the geometric properties of the lesions. This is particularly relevant for patients with one or few brain metastases, as well as those with benign brain lesions, where high conformity and a steep dose gradient outside the target volume are crucial to further minimize potential adverse events by limiting radiation exposure to the surrounding healthy tissues.^8^ Additionally, the risk of side effects such as neurocognitive decline,^9^ as well as radiation necrosis could be minimized through a more thorough selection of the treatment technique, in a group of patients who will likely receive several treatment series. The primary objective of this study is to provide radiation oncologists and physicists with information to support decision making upon geometrical findings, by evaluating the techniques of DCA and VMAT in the above mentioned context. Furthermore, we provide a novel practical tool to facilitate optimized therapy technique selection.

## 2 METHODS

### 2.1 Lesion characteristics

Patients treated between 2018 and 2021 at our institution were retrospectively screened. Eighty‐nine brain lesions in 24 patients were analyzed. Lesions with planning target volume (PTV) no larger than 4.3 cm^3^ were included. This roughly corresponds to a diameter of 2 cm for spherical lesions. Additionally, minimum distance of 2 cm from sensitive organs at risk (OAR) such as the brainstem, hippocampus, optic nerves, and chiasm was considered. This would help avoiding major dose modulation, which could generate bias. In favor of VMAT‐likewise, a minimum 5‐mm distance from the cranial vault was necessary to ensure that the V90% and V50% of the brain are not underestimated. The lesions included were stratified into four groups according to their volume: Category 1, lesions up to 1 cm^3^; category 2, 1–2 cm^3^; category 3, 2–3 cm^3^ and category 4, ≥3 cm^3^. To account for multiple testing, significance levels for correlation analyses between asymmetry parameters and conformity index (CI) were adjusted using the Bonferroni correction. For each volume group, five asymmetry parameters were tested, resulting in an adjusted significance threshold of *p*  <  0.01 (0.05/5) for statistical significance and *p*  <  0.004 (0.02/5) for high statistical significance. Nominal significance levels (uncorrected) were set at *p*  <  0.05 and *p*  <  0.02. The five asymmetry parameters represent related characteristics of the same lesion and are therefore not fully independent.

Comparing VMAT and DCA techniques across four different volume categories, significant differences were found for the CI. The number of cases per category were as follows: category 1 included 27 cases, category 2 included 26 cases, category 3 included 17 cases, and category 4 included 19 cases.

### 2.2 Treatment planning

Pre‐treatment contrast‐enhanced Magnetic resonance Imaging (MRI; Achieva, Philips Medical Systems, 1.5T, Netherlands) were acquired with a slice thickness of 1 mm, followed by a native planning Computer Tomography scan (CT; iCT SP, Philips Medical Systems, Netherlands) with a pixel size of 0.977 × 0.977 mm^2^ and 1 mm slice spacing. A thermoplastic stereotactic mask (Frameless SRS Mask Set cranial, Brainlab AG, Munich, Germany) was used for immobilization. The gross target volume (GTV) was defined on the T1‐weighted contrast‐enhanced sequence and rigidly registered to the planning CT scan. An isotropic 1 mm margin was added to create the PTV. Positional verification and correction were carried out using the Brainlab ExacTrac X‐Ray 6D Systems (Brainlab AG, Feldkirchen, Germany). All patients were treated using a True Beam STx (Varian Medical Systems, Palo Alto, CA, USA) equipped with an HD MLC120. The central 32 leaves of both MLC banks had a thickness of 2.5 mm. For patients with multiple metastases, separate plans were created for each metastasis, with the isocenter defined at the geometric center of each metastasis. The treatment planning software Eclipse‐Version 15.6.06 and PO‐Version 15.6.06 (Varian Medical Systems, Palo Alto, CA, USA) were used for dose distribution optimization. Dose calculation was performed using the analytical anisotropic algorithm (AAA) version 15.6.06, with a calculation grid of 0.125 cm. RT plans were generated using a single arc and three equidistant half arcs. For both the VMAT and DCA plans, the same field arrangement, collimator angle, and aperture were used. The collimator angles were adjusted individually, and the aperture extended 1 mm beyond the contour of the PTV. The field geometry for each of the arcs was selected as follows: Arc1 with a table position of 0°, extended from 150° to 210°; Arc2, with a table position of 90°, ranged from 190° to 350°; Arc3, with a table position of 45°, spanned from 200° to 340°; and Arc4, with a table position of 315°, covering the range from 160° to 20°. The treatment planning was carried out using 6 MV photon energy and high dose rates (up to 1400 MU/min) in the Flattening Filter‐Free (FFF) mode without a compensating filter.

Depending on clinical practice, all lesions were irradiated with an SRS dose of 20 Gy or fractionated stereotactic treatment with 3 × 9 Gy. For comparability, SRS plans with 20 Gy were also created for the latter. All plans for both techniques were normalized to ensure that 99.5% of the volume received 100% of the prescribed dose. In the optimization of VMAT plans, a maximum dose of 25 Gy was allowed. For the DCA plans, field weights were manually adjusted to achieve a minimum V_20Gy_ for the brain structure while maintaining the chosen normalization.

### 2.3 Assessment criteria for the plans: CI, spillage, and differences in percentage

Given the consistent normalization of all plans, the CI according to RTOG (Equation 1) was chosen to evaluate the conformity of the calculated RT plans. This index is calculated as the ratio of the volume receiving the prescribed dose (PIV) to the (planning) target volume (TV).

(1) $$\mathrm{CI}\;=\frac{\mathrm{PIV}}{\mathrm{TV}}\;$$

An optimal value for the CI is ≤ 1.2, with values ≤ 1.4 considered acceptable.^10^ This index provides an insight into the distribution of the prescribed dose outside the target volume due to its inherent simplicity. Additionally, the Paddick conformity index (PCI) was calculated for completeness^11^, ^12^ while not providing any additional benefit since all plans have equal normalization. The dose spillage index (DSI) (Equation 2) determines the ratio of the volume of energy dose distributed outside the TV (V_x%_‐TV) to the TV.^12^

(2) $$\mathrm{DS}I_{x\%}=\frac{V_{x\%-}\mathrm{TV}}{\mathrm{TV}}\;$$

The DSI is also used for categorizing the dose spillage volume into high (*x* = 90%), medium (*x* = 50%), and low (*x* = 25%). To enhance the presentation of results, a CI‐difference (ΔCI_%_) (Equation 3) and a dose spillage volume‐difference (ΔDS_x_%) (Equation 4) in percentage were calculated between both techniques. The reference is the average of the CI or dose spillage for the respective lesions from both plans.

(3) $$\Delta CI\%\;=2\times \frac{V_{20\;\mathrm{Gy}\;\mathrm{VMAT}}-V_{20\;\mathrm{Gy}\;\mathrm{DCA}}}{V_{20\;\mathrm{Gy}\;\mathrm{VMAT}}+V_{20\;\mathrm{Gy}\;\mathrm{DCA}}}\times 100$$

(4) $$\;\Delta DS_{x\%}=2\times \frac{V_{x\%\mathrm{VMAT}}-V_{x\%\mathrm{DCA}}}{V_{x\%\mathrm{VMAT}}+V_{x\%\mathrm{DCA}}-2\mathrm{TV}}\times 100$$

A positive value indicates a higher CI and DSI with VMAT, while a negative value suggests a higher CI and DSI with DCA. This approach allows for a detailed analysis of CI and Dose spillage differences for individual lesions and the identification of variations between the techniques. Effects such as larger CI for smaller volumes or increased Dose spillage for larger volumes could be minimized.

### 2.4 Determination of asymmetry parameters for the irradiated lesions

In this study, five specific parameters were used to describe and investigate asymmetry.

**Roundness**: The ratio of the mean curvature radius of convex areas within the lesion to the radius of the lesion, calculated using formula (Equation 5).

(5) $$R=\frac{\sum_{1}^{N}r_{i}/N}{R_{incircle}}\;$$

Where *R*_incircle_ represents the radius of the inscribed circle, and r_i_ represents the curvature radii of convex areas within particle *i*. To determine roundness, the medical open‐source software 3D Slicer 5.2.2. was used.^13^

**Sphericity**: Using the same software, the volume and surface of the lesions were calculated based on the DICOM data. With this information, ψ was determined as the ratio of the surface area of a volume‐equivalent sphere to the actual surface area of the lesion, calculated as follows (Equation 6):

(6) $$\psi \;=\frac{\sqrt[{3}]{36\pi V^{2}}}{A_{0}}\;$$

Where *V* represents the volume, and *A*_0_ represents the surface area of the lesion. A lesion with a spherical geometry has a sphericity of 1, whereas a flat disk would have a sphericity close to 0.^4^

**Sphericity of an Ellipse (ψEllipse)**: To spare physicists/radiation oncologists from using external software, an approach was employed to calculate sphericity based on the respective axis diameters of the lesions. Assuming that the lesion has an ellipsoidal shape, the axis diameters were determined according to the Response Evaluation Criteria in Solid Tumors 1.1^14^: The longest diameter of the PTV (a) and its perpendicular diameter on the transverse plane (b) were measured, and then the longest dimension perpendicular to the transverse plane (c) was determined.

With the determined axis diameters, the surface area of an ellipsoid could be calculated. This calculation was based on the Ramanujan formula^15^ for the surface area of an ellipsoid, denoted as *A*_0_ (Equation 7).

(7) $$\;A_{0}=\;\pi \sqrt{\frac{a^{2}b^{2}+a^{2}c^{2}+b^{2}c^{2}}{3}}$$

Furthermore, the volume of the ellipsoid was calculated, which can be determined using the following formula (Equation 8):

(8) $$V=\frac{\pi }{6}\;abc$$

Subsequently, the sphericity of the ellipse, which represents the ratio of the surface area of a sphere with the same volume to the surface area of the ellipsoid, was calculated. The sphericity ψ_Ellipse_ is determined using the following formula (9):

(9) $$\psi_{\mathrm{Ellipse}}=\frac{\sqrt[{3}]{a^{2}b^{2}c^{2}}}{\sqrt{\frac{a^{2}b^{2}+a^{2}c^{2}+b^{2}c^{2}}{3}}}\;$$

With these formulas and the axis diameters, the sphericity of each PTV could be directly calculated.

**Description of asymmetry through the Feret Axis Max_D_**: The asymmetry of a lesion can also be determined using the asymmetry quotient Q_asym_, which we propose. Q_asym_ relates the maximum diameter of the lesion, Max_D_, which can be directly determined in the planning software, to the effective diameter derived from the TV. Max_D_ represents the greatest distance between two points on the edge of the lesion, and the effective diameter is equal to the diameter of a sphere with the same volume as the TV. The latter can be calculated with the formula $1.2407\times \sqrt[{3}]{\mathrm{TV}}$. Q_asym_ can be calculated as follows:

(10) $$Q_{\mathrm{asym}\;=\frac{\mathrm{Ma}x_{D}}{D_{\mathrm{eff}}}\;\approx \frac{\mathrm{Ma}x_{D}}{1.2407\times \sqrt[{3}]{\mathrm{TV}}\;}}$$

**PTV_asym%_ according to Lee et al**.^16^: The axis diameters, which were previously used to calculate sphericity, were utilized for this purpose. The diameter measurements were carried out following the RECIST guidelines described above. From these measurements, PTV_asym%_ was calculated using the following equation (Equation 11):

(11) $$\mathrm{PT}V_{\mathrm{asym}\%}=\frac{\left|a-\right.\left.m\right|+\left|b-\right.\left.m\right|+\left|c-\right.\left.m\right|}{4m}\;\times 100$$

In this equation, *a*, *b*, and *c* represent the axis diameters of the PTV, and *m* is the mean of *a*, *b*, and *c*. The number “4” in the denominator serves as a normalization factor to scale PTV_asym%_ from 0% (most symmetric) to 100% (most asymmetric).

### 2.5 Data analysis and statistics

Data analysis was conducted using IBM SPSS Statistics Version 27 (IBM Corporation, Armonk, NY, USA). In an initial step, violin‐box plots were created to visualize the DSI, CI, and MUs for both applied techniques in each volume category. To analyze differences between the two techniques across volume categories and metrics (CI, DSI), the unpaired *t*‐test was employed. Within each category, a Spearman correlation analysis was additionally performed to determine the relationship between CI, DSI_x%,_ and the various asymmetry parameters.

## 3 RESULTS

### 3.1 Descriptive statistics of lesions

**FIGURE 1:**
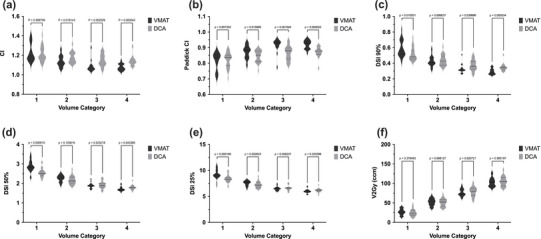
Detailed analysis of differences between DCA and VMAT using violin diagrams regarding (a) CI, (b) Paddick CI, (c) DSI_90%_, (d) DSI_50%_, (e) DSI_25%_, and (f) V_2Gy_ for volume categories 1–4. The corresponding *p*‐values for each volume category are provided. CI, conformity index; DSI, dose spillage index; VMAT, volumetric modulated arc‐therapy.

For volume category 1, no significant difference between the techniques was observed (*p* = 0.809). However, volume categories 2–4 showed significant differences with *p*‐values of 0.019, 0.004, and 0.001, respectively. In category 3 and 4, VMAT demonstrated superior conformity with significantly lower CI values than the DCA technique (Figure 1). The analysis of DSI at various percentages (90%, 50%, 25%) and V_2Gy_ across all volume categories also showed significant differences:

**DSI_90%_**: In volume category 1, DSI was lower in DCA (*p* = 0.019), while in volume categories 3 and 4, VMAT achieved lower values (*p* = 0.013 and *p* = 0.001).

**DSI_50%_**: In volume category 4, VMAT exhibited lower spillage (*p* = 0.007), while in volume category 1, DCA achieved better results.

**DSI_25%:_** DCA was superior in volume category 1 with significantly lower values (*p* < 0.001).

**V_2Gy_**: In volume category 1, DCA showed lower values (*p* = 0.001).

Overall, the results indicate that VMAT demonstrates better conformity (measured by CI) and lower high dose and medium DSI values in volume categories 3 and 4, while DCA exhibits superior results in volume category 1, as indicated by DSI and V_2Gy_ values.

**Correlation of CI with ψ, R, ψ_Ellipse_, Q_asym_, and PTV_asym%_ for DCA and VMAT**

**FIGURE 2:**
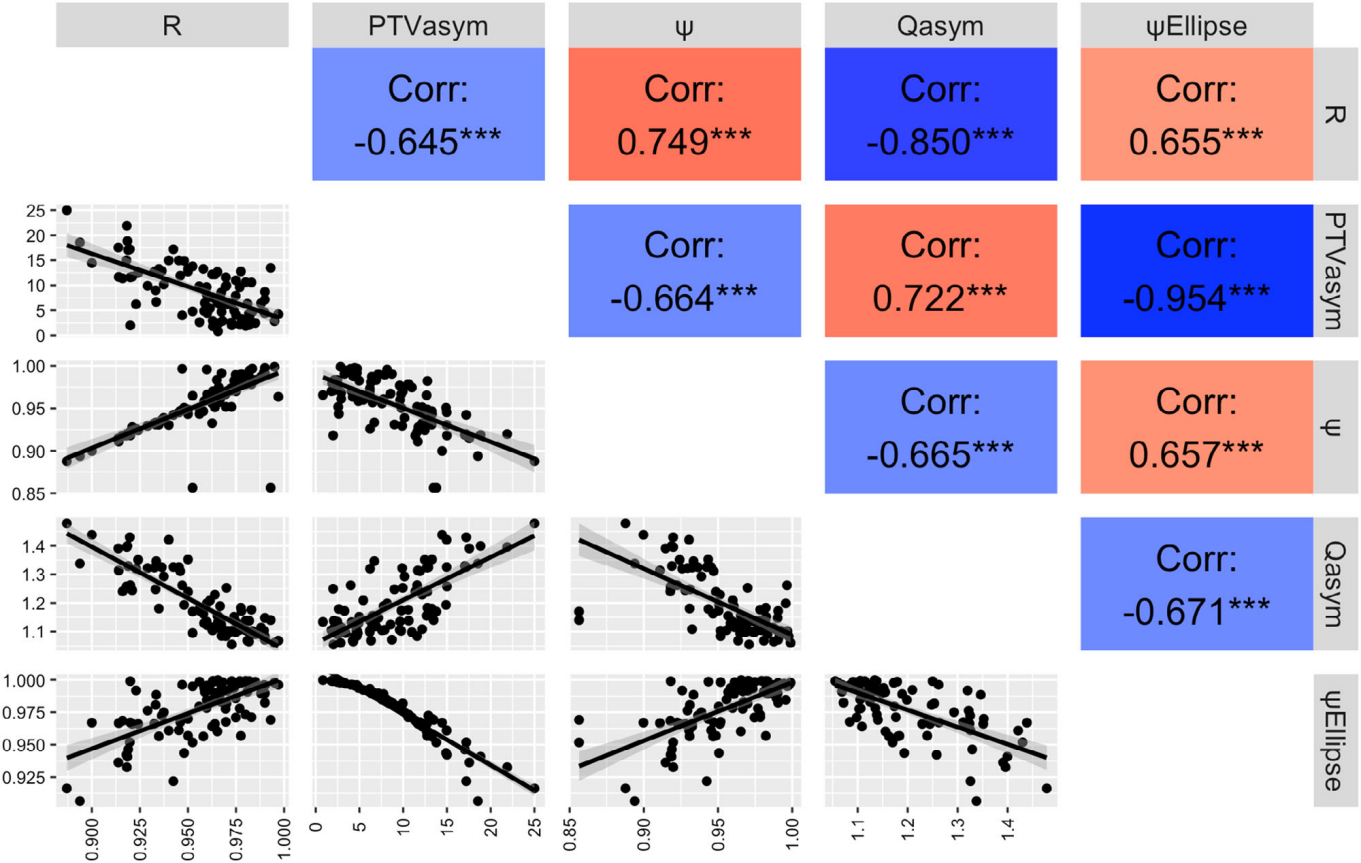
Correlation plot illustrating the relationships between the sphericity parameter R, ψ, ψ_Ellipse,_ Q_asym_, and PTV_asym%_. The upper triangular section shows the Pearson correlation coefficients. In the lower triangular section, scatterplots display pairwise relationships between variables. PTV, planning target volume.

Pearson correlations were calculated and evaluated between the various asymmetry parameters ψ, R, ψ_Ellipse_, Q_asym_, and PTV_asym%_ (Table 1, Figure 2) and CI_DCA_ as well as CI_VMAT_ for the four volume categories as previously described (Table 2). Significant correlations between Q_asym_ and CI_DCA_ were observed in all volume categories. This suggests that Q_asym_ has an impact on CI_DCA_ in all volume categories (*p* < 0.02). Other asymmetry parameters also showed significant correlations in specific volume categories. For instance, parameters ψ and R exhibited significant negative correlations with CI_DCA_ in volume categories 3 and 4 (*p* < 0.02). On the other hand, ψ_Ellipse_ and PTV_asym%_ exhibited weaker significance in some categories (*p* < 0.05), or showed no significant correlation. Q_asym_ displayed a significant correlation across all volume categories. Of note, CI_VMAT_ generally did not show significant correlations with the asymmetry parameters in any volume category (Table 2).

**TABLE 1:** Descriptive statistics for lesion asymmetry parameters.

	*N*	Min.	Max.	Mean	SD
ψ	89	0.86	1	0.9545	0.03081
R	89	0.89	1	0.9564	0.02538
ψ_Ellipse_	89	0.91	1	0.9776	0.02114
Q_asym_	89	1.05	1.48	1.1937	0.10731
PTV_asym%_	89	0.83	25	8.9087	5.16240

Abbreviation: PTV, planning target volume.

**TABLE 2:** a) Correlation of conformity index (CI) with asymmetry parameters for DCA across volume categories, b) Correlation of CI with asymmetry parameters for VMAT across volume categories.

a)				
Volume category	1	2	3	4
CI (DCA)				
ψ	−0.440	−0.362	−0.676^b^	−0.855^b^
R	−0.353	−0.393	−0.694^a^	−0.888^b^
ψ_Ellipse_	−0.487^a^	−0.242	−0.469	−0.372
Q_asym_	0.545^b^	0.432	0.593^a^	0.719^b^
PTV_asym%_	0.484^a^	0.286	0.433	0.465

b)
Volume category	1	2	3	4
CI (VMAT)				
ψ	0.389	0.203	0.133	−0.288
R	0.403	0.274	0.098	−0.131
ψ_Ellipse_	0.420	0.196	0.265	0.310
Q_asym_	−0.446	−0.187	−0.285	−0.207
PTV_asym%_	−0.477	−0.134	−0.324	−0.313

Note: Nominal significance levels (uncorrected) are *p*  <  0.05 and *p*  <  0.02.

^a^ Significant after Bonferroni correction for five comparisons per volume group (adjusted p  <  0.01).

^b^ Highly significant after Bonferroni correction for five comparisons per group (adjusted *p*  <  0.004).

Abbreviations: CI, conformity index; DCA, dynamic conformal arc; PTV, planning target volume; VMAT, volumetric modulated arc‐therapy.

**Examination of ΔCI% and ΔDS% as a function of Q_asym_**

**FIGURE 3:**
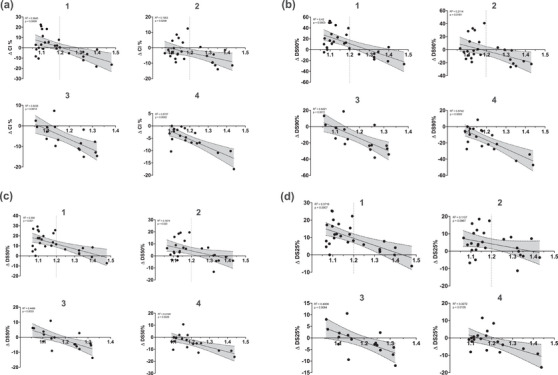
Scatterplot of (a) ΔCI%, (b) ΔDS_90%_, (c) ΔDS_50%_, and (d) ΔDS_25%_ as a function of Q_asym_ for volume categories 1–4. The horizontal line at zero serves as an indicator: positive values suggest lower CI, DSI_90%_, DSI_50%_, and DSI_25%_ in the DCA plans, while negative values suggest higher values for these parameters in the DCA plans. The corresponding R‐ and *p*‐values for the linear correlation between Q_asym_ and each Δ‐parameter are displayed within the respective subplots. CI, conformity index; DCA, dynamic conformal arc; DSI, dose spillage index.

Of particular note is the trend towards negative values for volume categories 3 and 4, as well as the negative values for categories 1 and 2 from a Q_asym_ of 1.2, especially in the high‐dose range (Figure 3).

### 3.2 Comparison of the DCA and VMAT

A median dose delivery of 4154.3 MUs for DCA and 6848.7 for VMAT was registered (*p* < 0.01). The interquartile range (IQR) was 675.45 for DCA and 1194.15 for VMAT, indicating higher variability in VMAT plans.

## 4 DISCUSSION

**FIGURE 4:**
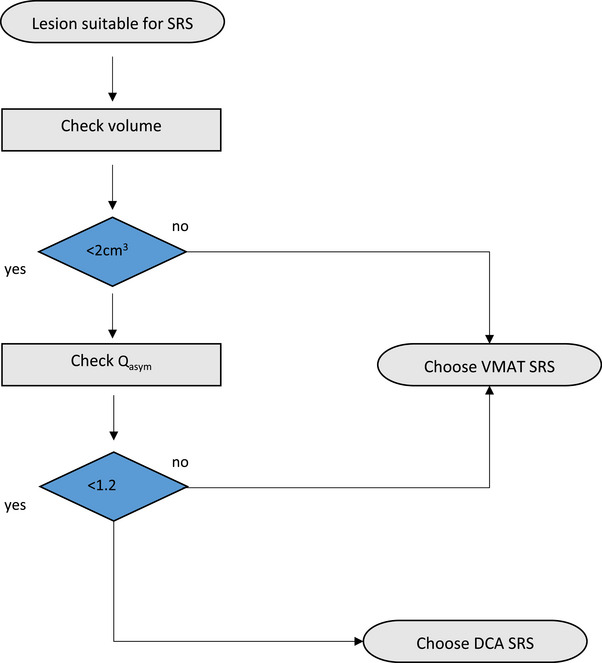
Suggested decision‐making SRS workflow. This flowchart assists in selecting the appropriate technique based on the volume and asymmetry of the lesion. SRS, stereotactic radiosurgery.

The results of our study provide important guidance for radiation oncologists and physicists in selecting the optimal technique for SRS of intracranial lesions. We propose the following approach: Depending on lesion size and proximity to critical OARs, suitability for SRS is assessed. Next, a treatment technique should be selected following the provided flow chart (Figure 4). For volume categories 1 and 2, when Q_asym_ values reach or exceed 1.2, the VMAT technique offers superior dose conformity and lower dose spillage compared to the DCA technique. For lesions with larger volumes (> 2 cm^3^, i.e., categories 3 and 4), the VMAT technique generally performs better, regardless of Q_asym_. When treating lesions < 2 cm^3^ with a Q_asym_ < 1.2, DCA and VMAT appear to be equivalent. Significant differences in CI between VMAT and DCA were particularly observed in volume categories 3 and 4. While no significant differences in CI were found in volume category 1, VMAT showed superior conformity in categories 3 and 4. Regarding DSI, the DCA technique outperformed VMAT in volume category 1, especially in DSI_25%_ and V_2Gy_, while VMAT showed lower spillage values in categories 3 and 4.

However, there are specific considerations that may justify a preference for DCA. DCA techniques are generally less complex since they are not intensity‐modulated, which may offer a faster, more efficient workflow and a simpler and less expedited verification process without the need for individual plan verification.^17^ Additionally, DCA plans are more robust due to their lower modulation, making them less susceptible to planning and treatment errors. DCA plans also require fewer MUs, sparing unnecessary dose exposure to the patient due to lower scattered radiation and a lower likelihood of dosing errors. It is essential to highlight that for lesions located in proximity to critical OARs, VMAT remains the optimal RT technique. In such cases, inverse planning is possible regardless of lesion size.^2^

Likhacheva et al.^18^ reported no predictive value for the number of lesions regarding OS, LC, and distant brain control. Lesion size, on the other hand, played a particularly important role, as patients with lesions < 2 cm^3^ showed significantly better OS. Yamamoto et al.^19^ found no significant difference in OS between patients with five to ten brain metastases and those with two to four brain metastases. However, patients with a singular metastasis had a superior OS, these results emphasize the need to optimize the management of smaller singular brain metastases.

As the number of fields and their geometric arrangement can strongly influence planning results,^20^ we decided to use identical geometries for VMAT and DCA assuring most accurate comparisons. This distinguishes this work from other studies, such as those by Paddick et al.^21^ and Molinier et al.^22^ who used various field geometries. The influence of asymmetry on treatment planning has been investigated previously^16^ with a focus on circular collimator arc (CCA) and DCA techniques. Therein, a superior target coverage was reported for DCA planning of SRS to brain metastases. However, CCA plans yielded lower volumes of normal brain tissue irradiated with 12 Gy or more (V_12Gy_) for lesions with a PTV_asym%_ of ≤6.12%. In lesions with a PTV_asym%_ of > 6.12%, DCA was found to be advantageous, with a lower CI and a V_12Gy_ comparable to that of CCA technique. In our study, similar correlations with PTV_asym%_ were observed, but the additional parameters and the novel parameter Q_asym_ were more significant. Additionally, our study examined lesion volumes up to 4.3 cm^3^, while investigation by Lee and Kim^16^ only considered volumes up to 1.4 cm^3^.

Another study Chea et al.^23^ focusing on the asymmetry of lesions compared different techniques for the stereotactic RT of brain metastases, included Gamma Knife and Multiplan Brainlab (MBM). The authors concluded both techniques can achieve high‐quality standards, but the geometric properties of the target volume have a significant impact on the plan quality (Sphericity Index). As a result, MBM 2.0 could be an alternative to Gamma Knife, especially for targets with a sphericity above 0.78.

However, this study has some limitations as all lesions were not in close proximity to critical OARs. Due to the steep dose fall‐off, a D_0.1_ cm^3^ of 2 Gy was never exceeded. A statistical comparative analysis of OAR doses between the two techniques was not conducted because the 2 Gy threshold was not exceeded in any of the plans, as this was beyond the scope of this study. The results in Figure 3 indicate a clear trend in dose differences relative to Q_asym_. However, the number of plans in the volume categories were insufficient for a detailed ROC analysis. Our group will run a phantom‐based study that could validate our current results and provide further insights into the applicability of VMAT and DCA techniques across different asymmetries and volumes.

## 5 CONCLUSION

Introducing a novel treatment parameter Q_asym_, we provide an easy‐to‐use guidance tool for optimal treatment technique selection. In direct comparison, DCA provides a more favorable dose distribution profile for small and symmetrical brain metastases, while VMAT provides dosimetric advantages for larger and more irregular volumes.

## AUTHOR CONTRIBUTIONS

**Lara Caglayan**: Responsible for drafting and revising the manuscript; conducted the data collection and analysis. **Davide Scafa**: Responsible for drafting and revising the manuscript. **Patrick Eich**: Review of the manuscript. **Christina Leitzen**: Review of the manuscript. **Fabian Kugel**: Review of the manuscript. **Stephan Garbe**: Review of the manuscript. **Christopher Schmeel**: Review of the manuscript. **Gustavo Renato Sarria**: Review of the manuscript. **Christos Moustakis**: Review of the manuscript. **Baltas Dimos**: Review of the manuscript. **Julian Philipp Layer**: Review of the manuscript. **Cas Stefaan Dejonckheere**: Review of the manuscript. **Andrea Glasmacher**: Review of the manuscript. **Shari Wiegreffe**: Review of the manuscript. **Anca L Grosu**: Review of the manuscript. **Eleni Gkika**: Review of the manuscript. **Youness Nour**: Conceptualized the study, designed the methodology, and conducted the data collection and analysis; responsible for drafting and revising the manuscript.

## CONFLICT OF INTEREST STATEMENT

The authors declare no conflicts of interest.

## ETHICS APPROVAL

This study was approved by the Ethics Committee of the Medical Faculty at the University of Bonn (Ethical Approval 2024‐105‐BO)

## Data Availability

Research data are stored in an institutional repository and will be shared upon request to the corresponding author.
